# A balanced solution to the cumulative threat of industrialized wind farm development on cinereous vultures (*Aegypius monachus*) in south-eastern Europe

**DOI:** 10.1371/journal.pone.0172685

**Published:** 2017-02-23

**Authors:** Dimitris P. Vasilakis, D. Philip Whitfield, Vassiliki Kati

**Affiliations:** 1 Department of Environmental and Natural Resources Management, University of Patras, Agrinio, Aitoloakarnania, Greece; 2 Hellenic Republic, Decentralized Administration of Macedonia-Thrace, Directorate of Evros Region Forestry Service, Alexadroupolis, Evros, Greece; 3 Natural Research Ltd, Brathens Business Park, Banchory, Aberdeenshire, United Kingdom; Universidade de Lisboa Instituto Superior de Agronomia, PORTUGAL

## Abstract

Wind farm development can combat climate change but may also threaten bird populations’ persistence through collision with wind turbine blades if such development is improperly planned strategically and cumulatively. Such improper planning may often occur. Numerous wind farms are planned in a region hosting the only cinereous vulture population in south-eastern Europe. We combined range use modelling and a Collision Risk Model (CRM) to predict the cumulative collision mortality for cinereous vulture under all operating and proposed wind farms. Four different vulture avoidance rates were considered in the CRM. Cumulative collision mortality was expected to be eight to ten times greater in the future (proposed and operating wind farms) than currently (operating wind farms), equivalent to 44% of the current population (103 individuals) if all proposals are authorized (2744 MW). Even under the most optimistic scenario whereby authorized proposals will not collectively exceed the national target for wind harnessing in the study area (960 MW), cumulative collision mortality would still be high (17% of current population) and likely lead to population extinction. Under any wind farm proposal scenario, over 92% of expected deaths would occur in the core area of the population, further implying inadequate spatial planning and implementation of relevant European legislation with scant regard for governmental obligations to protect key species. On the basis of a sensitivity map we derive a spatially explicit solution that could meet the national target of wind harnessing with a minimum conservation cost of less than 1% population loss providing that the population mortality (5.2%) caused by the operating wind farms in the core area would be totally mitigated. Under other scenarios, the vulture population would probably be at serious risk of extinction. Our ‘win-win’ approach is appropriate to other potential conflicts where wind farms may cumulatively threaten wildlife populations.

## 1. Introduction

Harnessing the kinetic energy of moving air is widely recognized as an environmentally friendly strategy to combat climate change, and an ambitious global target for humanity to satisfy 20% of its energy demands through wind power by 2050 has been recommended [1], requiring 50 fold more technological capture of wind power than present [2]. Capturing wind power is therefore currently the fastest growing source of renewable energy world-wide so that wind farm developments are flourishing [3]. Wind energy capture, however, can raise serious environmental concerns, and development versus conservation conflicts can arise, because wind turbines can have several adverse environmental impacts, including noise disturbance and modification of local weather [2,4].

One such impact of concern is mortality for species prone to collision with turbine blades, such as birds [5–11]. Old World vultures appear especially prone to collisions due to their flight behaviour, such that wind farms might collectively pose serious threat to populations [12–20].

The massive expansion of wind farm developments is a modern example of the well-known issue of the “tyranny of small decisions” [21], implying a major cumulative effect that a set of individually minor incremental effects can create when combined [22,23]. Acknowledging the importance of cumulative effects of potentially harmful development projects, such as wind farms, the European Union (EU) has formulated a relevant legislative framework. New wind farm proposals should accord with the Strategic Environmental Assessment (SEA) Directive, calling for sustainable spatial planning at a broad, often national, scale [24]. Furthermore, they should be subject at a site-specific level to Environmental Impact Assessment Studies (EIAs) requiring cumulative impact assessments, after another EIA Directive [25]. Additionally, when wind farm developments may potentially affect interests protected by the Natura 2000 network of classified sites, Appropriate Assessments are often required to ensure that it is beyond scientific doubt that they will not adversely affect the protected interests [26,27]. A recent review [28] has concluded that such legislation is often ignored in practice (see also [29–31]).

Our study area [20] is recognized as a priority area for bird conservation in hosting many Special Protected Areas (SPAs) within the Natura 2000 network [32], including, in particular, the sole breeding population of cinereous vulture (*Aegypius monachus*) in south-eastern Europe [33–35]. Much of it, however, is also designated as a wind farm priority area (hereafter WPA), according to the Greek SEA [36]. Our case study hence well illustrates an emerging generic conflict of industrialized wind energy development in ecologically sensitive areas, as evinced by the sharp increase of developers’ interest in future wind farms in our study area, when the currently operating wind farms alone account for a substantial population loss of cinereous vultures due to collision mortality [20]. As presaged by [20] the next challenge, within which our present study lies, therefore, is in sustainable spatial planning that balances future wind energy developments as a strategy to combat climate change, while minimizing their adverse cumulative impacts on wildlife.

Our particular objectives were: (a) to evaluate the cumulative collision mortality of wind farms on the cinereous vulture population, accounting for all operating and proposed wind farms, and (b) to provide a spatially explicit solution for sustainable spatial planning in practical terms, which would allow meeting targets for wind energy capture at minimal cost to the small and vulnerable vulture population.

In meeting these objectives we evaluate the implementation of the EU legislative framework regarding wind farm development impacts, and its effectiveness for cinereous vulture conservation. We also discuss the advantages of adopting our methodological approach when rapid conservation decisions are needed at broad scales, and when supporting data are often incomplete or absent at individual development sites.

## 2. Materials and methods

### 2.1 Study area

**Fig 1:**
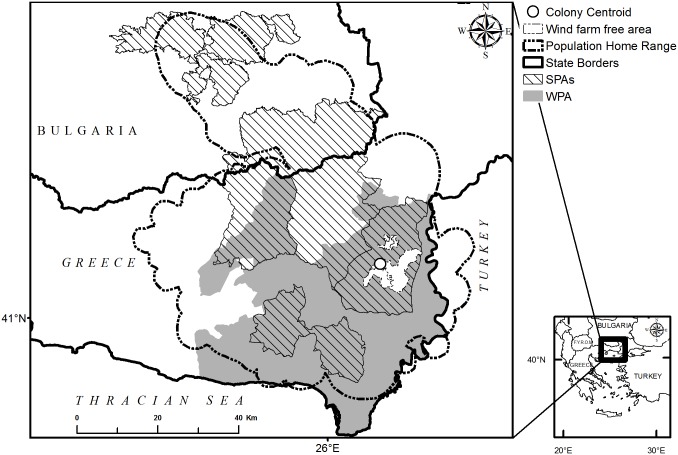
Spatial configurations of wind farm development, wind farm-free and cinereous vulture conservation areas. Special Protection Areas (SPAs) designated for the conservation of cinereous vulture under the Birds Directive (EC, 2009), wind farm free designated area of Dadia-Lefkimi-Soufli Forest National Park, and Wind farm Priority Area (WPA), within the population home range of cinereous vulture (after Vasilakis et al., 2016).

The study area is in the Eastern Rhodopes Mountains in the Balkans, covering an area of 15,000 km^2^ in Greece and Bulgaria (Fig 1). It is characterized by gentle topography (average altitude: 171 ± 117 m) and a Continental-Mediterranean climate. It is a thinly populated forested area of broad-leaved and pine woods, alternating with pastures and agricultural mosaics and has a remarkable biodiversity value, including 36 of the 38 European diurnal raptor species [34]. It includes 16 Special Protection Areas (SPAs) under the provisions of the Directive on the Conservation of Wild Birds ([32]: 10 in Greece and six in Bulgaria), with cinereous vulture being a protected qualifying interest in eight (Fig 1). A substantial part of the study area is also identified as one of the highest priority areas (Wind farm Priority Area, WPA) for harnessing wind energy in Greece, with a governmental target of establishing 480 wind turbines (960 MW) according to a Strategic Environmental Assessment Study [36].

### 2.2 Cinereous vulture population

The study area supports the sole cinereous vulture breeding population in south-eastern Europe, which is endangered in Greece and globally near-threatened with a decreasing population outside Europe [37,38]. It is a tree-nesting, non-territorial but wide-ranging semi-colonial species, behaving as a central-place forager when breeding [39]. The species breeds in Dadia-Lefkimi-Soufli National Park of Greece, having a relatively stable population with an average of 103 individuals including 24 breeding pairs (period 2004–2012). A proportion of 12.7% of the population (on average) were fledglings (1^st^ calendar year, CY), 11.6% were juveniles (2^nd^ CY), 20.3% were immature (3^rd^-4^th^ CY) and 55.5% were adults (>5^th^ CY) [35,40] (Skartsi Th., *pers*. *comm*.). The population home range is 4970 km^2^ of which 39% (1942 km^2^) is the population core area, where individuals maximize their activity [20].

### 2.3 Background datasets

To depict prospects for wind energy developments, and their possible influence on our study species, we considered spatial data for the WPA; those SPAs including cinereous vulture as a designated interest, as well as the estimated home range of the cinereous vulture population [20]. We also calculated these features’ spatial overlap (Fig 1). To assess collision mortality, we used a sensitivity map for cinereous vulture [20], and two additional datasets: the first comprised the technical characteristics of the operating and proposed wind farms in the study area and the second referred to the flight activity of cinereous vultures in the same area. Further details of these datasets follow.

#### Wind farm dataset

A broad prospective for wind farm development was given by the Greek government, for much of our study area, to establish 480 wind turbines (960 MW) [36]. From national planning systems we accessed, where possible, the coordinates and number of turbines and their technical characteristics for 155 wind farm projects (1712 turbines), including 13 operating wind farms (185 turbines, 253.2 MW) (S1 Table). For all projects, exact turbine coordinates and their technical characteristics were missing for 22.8% and 24.3% projects respectively (S1 Table). For Greece, the planning stage per project was determined (submission, technical approval, environmental approval, operating: 137 wind farms, 1580 turbines), excluding those projects that had been rejected (from 2003 to 2013, 16% of projects were rejected at the technical approval stage; none were rejected at the environmental approval stage) [41]. For Bulgaria, all wind farm projects within the study area were classified as submitted, since no further planning information was available (18 wind farms, 132 turbines, Stoychev St., *pers*. *comm*.).

#### Cinereous vulture data

The second dataset comprised 14,822 locations stemming from seven cinereous vultures that were tagged with battery-powered Global Position System (GPS) units [20]. Vultures were tracked for 42% of the monitoring time as adults (>5^th^ calendar year, CY), 53% as immature (3^rd^- 4^th^ CY), and 5% as juveniles (2^nd^ CY) for the period 2007–2009 (S2 Table). The seven birds used in the analysis were not sexed, since both sex and age class do not affect cinereous vulture home range extent and space use in our study area, on the basis of the results of movement ecology study, analyzing the space use patterns of 12 individuals radio-tracked for 316 bird-months [42].

Specific permission for the field study (trapping and tagging the vultures) was given by the Directorate of Aesthetic Forests, National Parks and Game Management of Ministry of Environment Energy and Climate Change (Greece). Permission for ringing and tagging the vultures was given by the Hellenic Ringing Centre. Ethical approval of all sampling procedures and manipulations were given as part of obtaining the above mention licenses. The vulture handling time was minimized and all individuals were released immediately after processing. Transmitters were attached [43] as backpacks (transmitters plus harness) and weighed less than 3% of each vulture’s body mass [20] and no excoriations or other type of injury were observed on vultures whose backpacks were later removed due to non-transmission.

**Fig 2:**
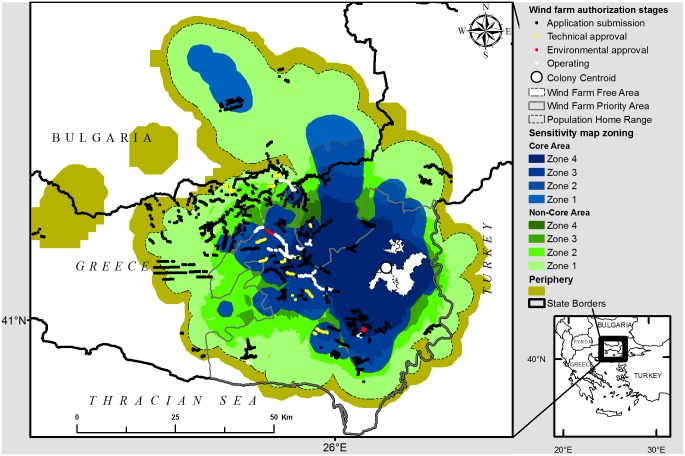
Wind farms at different authorization stages within a sensitivity map for cinereous vulture. Large numbers of wind farms are concentrated in areas of vital conservation importance (70% of time spent by individuals on average), as indicated by nine zone sensitivity map for cinereous vulture (*Aegypius monachus*) (from Vasilakis et al. 2016).

This dataset comprised three-dimensional coordinates, including flight height above sea level for each location, with a positional dilution of precision ≤ 10m (mean linear location error ≤ ± 9.1 m, SE = 0.15) involving 70 bird-months (years 2007–2009) (S2 Table). We also used a sensitivity map for the cinereous vulture population, based on a conservation prioritization system of nine zones [20]. This includes a core area of vital importance (70% of time spent by individuals on average), a non-core area and a periphery (less than 5% of time spent). The core and non-core areas are further prioritized in four conservation zones each, according to the population fraction that used each zone (1: <25%, 2: <50%, 3: <75%, 4: >75%) on the basis of a home range analysis of 19 individuals (7 GPS and 12 VHF tagged vultures) [20] (Fig 2).

### 2.4 Collision mortality estimation

We estimated the annual collision mortality for all wind farms (operating and planned) for each of the nine conservation zones of the sensitivity map described elsewhere [20]. We largely followed the same methods that were applied to estimate the collision mortality for each of the operating wind farms in the study area [20], which combined the Fixed Kernel method (population utilization distribution estimation) with a ‘Band’ Collision Risk Model (CRM) [44] at a resolution of 200 x 200 m pixels.

Otherwise, previous methods [20] were modified as follows: first, all wind farms within the same conservation zone were unified (thereby effectively considered as one ‘wind farm’); and therefore large scale collision mortality was extracted per conservation zone, and not per wind farm. Second, to extract a representative population fraction that used each conservation zone (zone 1 = 2/19, zone 2 = 7/19, zone 3 = 12/19 and zone 4 = 17/19), we calculated the median value of the four population quartiles accordingly defined by [20] (S3 Table). Third, to estimate the percentage of time the vultures fly at collision risk height of pooled wind farms per conservation zone, we used the average rotor swept heights of all wind farms per conservation zone (S3 Table) and the frequency distribution of vultures’ flight heights over ridges. The latter was calculated by subtracting the topographic elevation a.s.l. from the vultures’ flight height a.s.l. (provided by GPS) for the GPS fixes located over ridges of the study area (ridges buffered with 200 m radius, 3126 GPS fixes). The output was inserted as a metric in the CRMs [20,44].

When turbines’ coordinates were missing and only the polygon of the proposed wind farm’s footprint was known, we virtually located turbines on ridges falling in the proposed polygon at distances that equalled three times the corresponding turbine rotors’ swept diameter, as specified in national legislation [36]. When turbine technical metric values were missing, we took the average values of the turbines’ technical characteristics in the conservation zone where the turbine was proposed, since collision mortality estimation was performed per conservation zone.

As the avoidance rate is a highly influential component of the Band CRM’s outputs [45], we deployed four avoidance rates (95%, 98%, 99%, and 99.5%) in our CRM runs, following [20]. The final output was the estimated annual collision mortality per conservation zone for the four avoidance rates considered. We primarily report results from CRMs deploying a 99% avoidance rate. This choice is justified through a model validation [20], which compared predicted annual mortalities for the operating wind turbines [20] with the results from daily carcass searches (12 months; 2009–2010) in a 50 m buffer around 47% of the operating turbines, in the study area [46].

Finally, we calculated the cumulative collision mortality for operating and for all wind farms (operating and planned) per conservation zone, by removing each time the fraction of the population having already collided in a previous conservation zone, before calculating the collision mortality in the next zone in order, using Eq (1) [47], where *C*_*t*_ is the total cumulative mortality, *P* is the population size (*P* = 103 individuals), and n = total number of conservation zones.

$$C_{t}=\sum_{j=1}^{j=n}C_{j}\left\lfloor \frac{P-\sum C_{j-1}}{P}\right\rfloor$$ (1)

Hence, the cumulative collision mortality was calculated for each conservation zone (n = 9), from the most important zone of the core area up to the periphery (Fig 2). Our zonal approach for all wind farms (operating and planned) was undertaken in recognition of some uncertainties in the exact spatial nature of future proposals.

For reasons of comparison with our preferred cumulative mortality estimation method [47], we also calculated a simple additive mortality per zone for operating and all wind farms (operating and planned), by progressively summing collision mortalities for each zone.

Cumulative mortality was also calculated for the operating wind farms where spatial details were certain, on a case-by-case basis (n = 13), prioritizing them first by conservation zone and then by their distance from the breeding colony (distance measured from geometric centres of wind farms and breeding colony). Annual estimated mortalities for each operating wind farm were taken from [20]. We undertook this further calculation, for operating turbines, to gain insight, using ‘real’ data, into how our zonal approach may affect future cumulative mortality estimates. The result of this calculation, therefore, was contrasted with those from the zonal approach for these wind farms.

Finally, following this calculation, we also evaluated two more possible scenarios on potential vulture mortality. The first scenario assumed that 16% of turbines currently in the planning system, but not operational or having been decided on for approval would be rejected at the technical approval stage (based on the rejection rate already recorded 2003–2013: see above). The second scenario assumed that when the national target of nominal power (960 MW) for the WPA was met further wind farm development would stop.

## 3. Results

### 3.1 Wind farm development

Most wind farm projects were at the application stage (projects submitted account for 82% of overall nominal power in planning systems). Fewer projects had gained technical-economic feasibility approval (7% of overall power) or environmental approval following impact assessment study (1% of overall power). Currently operating wind farms accounted for only 9% of the nominal power within the planning system (Table 1 and S1 Table). In the worst-case scenario for potential impacts on vultures, if all proposed turbines are authorized, the number of turbines will be almost nine times higher than currently and would harness a nominal power of 2744 MW, exceeding by far the national target set for the WPA (960 MW) (Table 1 and S1 Table). Furthermore, all wind farms that have received environmental approval are located in the vulture population’s core area, and 45% of total power (proposed and current wind farms) is located in the core area, with only 8% in the periphery (Table 1 and Fig 2).

**Table 1:** Wind farm power of wind farms per conservation zone at different planning stages, in the vulture population’s core area, non-core area and periphery.

Conservation zones			Planning stage									
			Submission		Technical approval		Environmental approval		Operating		Total	
	Code	A (km^2^)	P (MW)	Tu	P (MW)	Tu	P (MW)	Tu	P (MW)	Tu	P (MW)	Tu
**Core area**	4	794	329	257			36	21	134	87	500	365
	3	540	293	190	89	44	26	13	72	57	478	304
	2	353	220	118	11	5	–	–	13	12	244	135
	1	255	–	63	–	–	–	–	–	–	–	63
	Subtotal	1 942	842	628	100	49	62	34	219	156	1223	867
**Non-core area**	4	26	4	4	–		–	–	–	–	4	4
	3	174	46	40	–		–	–	–	–	46	40
	2	593	350	208	–	7	–	–	13	10	377	225
	1	2235	815	415	52	26	–	–	21	19	888	460
	Subtotal	3 028	1215	667	66	33	–	–	34	29	1314	729
**Periphery**		1 580	207	116	–	–	–	–	–	–	207	116
**Grand Total**		6550	2263	1411	166	82	62	34	253	185	2744	1712

Code: 1: <25%, 2: 25–50%, 3: 50–75%, 4:>75% of population using each zone, A: zone area, P: power, Tu: Number of turbines.

SPA classifications which register the cinereous vulture as a qualifying interest cover 46% of our estimated population home range, as well as its breeding colony as a strictly protected wind farm-free area (that occupies 3.4% of ‘cinereous vulture’ SPAs, and 1.6% of population home range) (Fig 1). The Greek WPA (2276 km^2^) largely falls within the vulture population home range (91%) and half of it falls within the population core area (53%). About half of the WPA (49%) also coincides with SPAs classified for cinereous vulture, so that the number of wind turbines in SPAs could be anticipated to increase from the 129 currently operating turbines up to 801 turbines under the WPA.

### 3.2 Wind farm-originated mortality

If all turbines in the planning system were to operate simultaneously, the predicted cumulative annual collision mortality would account for 50% of the current standing population (under 99% avoidance rate: 51 deaths out of 103 individuals, 48 deaths in the core area; Table 2). As expected, the CRM avoidance rate choice heavily influenced outputs. Under a 99.5% and a 98% avoidance rate, the cumulative collision mortality was estimated to be 28 and 82 deaths respectively for the standing population, whereas under a 95% avoidance rate the population was driven to extinction by proposed turbines in only the fourth zone of the core area (the conservation zone closer to the colony), implying a rapid extinction in less than a year (Fig 2 and S4 Table). Regardless of the avoidance rate, a pattern emerged: (a) collision mortality was expected to be eight to ten times greater than mortality created by the currently operating wind farms and (b) more than 92% of expected deaths would occur in the core area of the population (Table 2 and S4 Table).

**Table 2:** Predicted additive and cumulative collision mortality per year for a cinereous vulture population (103 individuals) in the Balkans, stemming from (a) operating and (b) all proposed and operating wind turbines across a nine-zone conservation prioritization zoning system (see Vasilakis et al. 2016), with the help of CRM (99% avoidance rate).

Conservation zone		(a) Operating turbines								(b) Total turbines (proposed and operating)				
		C	C (%)	C_A_	CCz	CCw	D_AZ_ (%)	D_AW_ (%)	D_ZW_ (%)	C_b_	C_b_ (%)	C_A_	CCz	D_AZ_ (%)
**Core area**	4	3.76	67.27	3.76	3.76	3.76	0.00	0.00	0.00	38.21	62.46	38.21	38.21	0.00
	3	1.62	28.89	5.38	5.32	5.25	1.10	2.42	1.29	12.78	20.89	50.99	46.25	10.25
	2	0.13	2.27	5.50	5.44	5.37	1.19	2.49	1.26	4.01	6.56	55.01	48.28	13.94
	1	0	-	5.50	5.44	5.37	1.19	2.49	1.26	0.23	0.37	55.23	48.38	14.16
	Total	5.50	98.42	5.50	5.44	5.37	1.21	2.49	1.26	55.23	90.29	55.23	48.38	14.16
**Non-core area**	4	0	-	5.50	5.44	5.37	-	-	-	0.36	0.59	55.59	48.55	14.50
	3	0	-	5.50	5.44	5.37	-	-	-	1.58	2.58	57.17	49.28	16.02
	2	0.04	0.65	5.54	5.47	5.40	1.23	2.51	1.26	3.06	5.00	60.23	50.64	18.94
	1	0.05	0.93	5.59	5.52	5.45	1.25	2.53	1.27	0.75	1.22	60.97	50.95	19.68
	Total	0.09	1.58	5.59	5.52	5.45	1.25	2.53	1.27	5.74	9.39	60.97	50.95	19.68
**Periphery**		0	0	0.00	0.00	0.00	0.00	0.00	0.00	0.20	0.33	61.17	51.03	19.69
**Grand Total**		5.59	100	5.59	5.52	5.45	1.25	2.53	1.28	61.17	100	61.17	51.03	19.88

Conservation zones 1: 1–4, 2: 5–9, 3: 10–14, 4: 15–19 individuals, C: Annual collisions, C (%): Percentage of annual collisions, C_A_: Additive annual collisions, CCz: Cumulative annual collisions at zone level (all wind farms pooled as one mega wind farm per zone), CCw: Cumulative annual collisions at operating wind farm level, D_AZ_: Overestimation difference [D_AZ_ = ((C_A_–CC_Z_)/CC_Z_)*100], D_AW_: Overestimation difference [D_AW_ = ((C_A_–CC_W_)/CC_W_)*100], D_ZW_: Overestimation difference [D_ZW_ = ((C_A_–CC_ZW_)/CC_ZW_)*100], C_**b**_: Annual collision per zone (all wind farms pooled as one mega wind farm per zone), C_**b**_ (%): Percentage of annual collisions.

### 3.3 Collision mortality minimization

To minimize expected mortality, only proposed turbines located in the two outer zones of the sensitivity map (periphery and part of the first zone of the non-core area), should be permitted to operate under environmental approval provisions, namely 576 turbines covering 55.41 km^2^ (Table 1 and S3 Table). In this case, the 960 MW national target of wind harnessing would be met (1095 MW nominal power), while the predicted collision mortality from these 576 turbines would be less than 1% of the standing population (Tables 1 and 2, Fig 2). This mortality refers only to the effect of future wind farms and so assumes that the mortality caused by the currently operating wind farms would be totally mitigated.

### 3.4 Cumulative mortality estimation

Our contrast of predicted collision mortality collectively from simple additive estimates with our preferred cumulative mortality estimation method (Eq (1), zonal approach) revealed higher estimates by the additive approach, ranging from 1.25% higher for the 13 operating wind farms up to almost 20% higher for all 155 proposed wind farms, depending on the number of projects considered.

For operating wind farms the difference in cumulative collision mortality estimation when utilising mortality estimates sequentially from every wind farm in Eq (1), rather than pooling these estimates under each of the nine conservation zones, resulted in a value 1.28% higher for the zonal approach (Table 2). Transferring these results for operating turbines to the whole suite of potential wind farms, then our zonal approach to considering future mortalities might lead to an overestimation, by about 12% in cumulative mortality calculations. Thus, correcting for this possibility, the predicted cumulative annual collision mortality for all turbines in the planning system could account for 44% of the current standing population (45 deaths out of 103 individuals).

If, as in 2003 to 2013, 16% of all the turbines (1712) awaiting approval in the planning system would be rejected at the technical approval stage, then 1486 turbines (including the operating number) would be installed. Based on the standing population of 103 individuals, this would cause 44 deaths (under a CRM 99% avoidance rate) accounting for 39% of the population.

Finally, if development stops when the national target (960 MW) for the WPA was met, 599 turbines (including the operating) would be installed, resulting in a cumulative collision mortality that would account for 17% of the standing population.

## 4. Discussion

### 4.1 Industrialized wind farm development

Our study has uncovered a potential problem of severe magnitude for biodiversity, generated by a rapid trend for large-scale industrial wind-farm proposals in the study area which could have profound consequences for south-eastern Europe. Undoubtedly, it is legislatively imperative for Greece to meet the targets set by the National Renewable Energy Action Plan, namely the 20% target of gross energy consumption stemming from ‘green energy’ by 2020 (63.9 TW) [48][49]. This ambitious target dictates a rapid increase of wind power capacity (from 1.6 GW in 2011 to 7.5 GW in 2020) and the use of wind farm energy is seen as the main renewable energy source (57% of overall ‘green energy’ capacity). This government target has understandably been associated with a rapid increase in wind farm developers’ interest, with our study area being a primary focus of such interest [36].

Given the expected sharp increase of wind farm developments in the study area in the near future, the nationally-derived Greek target within our study area (960 MW) will be exceeded by three fold, if all proposed projects were authorized. Of course, not all wind farm proposals placed into planning systems gain authorization, for various reasons. What is of serious concern, nevertheless (see later), is that the guiding instrument for wind farm developers in Greece, the WPA [36], paid scant regard to environmental impacts, even to SPAs; and that there has been no sign that potentially adverse environmental effects of wind farms have influenced authorization of proposals.

A real challenge therefore lies in influencing government policy through a balanced and objective approach to manage and spatially guide developers’ interest in a way that a positive cost-benefit ratio is gained in favour of both economic developments that hamper further global warming, and biodiversity conservation, within all relevant legislative frameworks [27]. Addressing this challenge was a primary objective of the present study, focussing on a key species of conservation concern, the cinereous vulture.

### 4.2 Methodological insights

In this study we combined spatial modelling [50] and a Collision Risk Model [44] to predict collision mortality for both operating [20] and planned wind farms at larger spatio-temporal scale. This approach, considering the collective effect of wind turbines on vulnerable raptor populations at a broad scale, is recognized as more biologically meaningful than the usual approach of case-by-case assessments [16,51]. Collision mortality prediction can be a particularly complex issue, since it is known that there is no a linear relationship between the frequency of observed birds in a wind farm area and the recorded fatalities [16]. The exact location of each wind turbine, the local conditions of topographical features and wind patterns that can affect bird use, can heavily influence collision mortalities, and so can account for large differences in fatalities among wind turbines, even within the same wind farm [16,18,52]. Our methodological approach integrates the effect of topography and prevalent wind patterns through the parameter of the proportion of time that vultures spent in an actual or prospective turbine area, instead of simply documenting the number of observed birds. On the other hand, our methodology was applied over a large area and concludes to mortality assessments over broad conservation zones, as a first and quick guideline for spatial planning of forthcoming wind farm industrial development. At this spatial scale and as a broad but reasonable guideline, nevertheless, other studies infer its merit [14]. Its merits would be even greater and assessments would be further refined, if the method was applied at finer scale, assessing mortality per turbine since CRMs, if populated by adequate flight data, can readily predict turbine specific fatality rates [16,18].

Our model was validated [20] on the basis of carcass survey data collected at about the same period (2009–2010) with cinereous vulture telemetry data around 47% of the operating wind turbines, reporting one cinereous vulture as victim of collision [20,46,53]. Since then data from systematic post-construction carcass survey of the operating wind turbines are lacking. The results from surveys and random incidences since 2008 conclude so far fatalities from collision for nine raptor species (17 carcasses), out of which two cinereous vultures, whilst recently (2015) a griffon vulture (*Gyps fulvus)* was found buried under rocks in the base of a wind turbine evincing a likely underestimation error, even from independent carcass surveys [46,53,54] (WWF Greece, Skartsi Th. *pers*. *comm*.). We suggest that a post-construction carcass survey in the study area and in other parts of the world would further contribute to model validation and calibration, when predicting mortalities with our method.

Some simple extrapolations of site-based mortalities to the future or to broader scales (e.g. [55]) which do not properly integrate future wind farms’ technical specifications and their spatial configuration [11], or populations’ demographic consequences cannot be conceived as cumulative mortality estimates because, apart from other problems, they ignore multiple sequential collision encounters with wind farms [56]. Our results confirm the overestimation of cumulative mortality by simple additive methods and argue further that the algorithm Eq (1) we primarily employed [47] is more appropriate.

Our approach can be criticised in that it was by definition static. We had to assume that future wind farms would operate simultaneously, as their real operation time remains unknown. Non-simultaneous operation, however, would only protract the collective impact, not dilute the overall impact; as we have documented, this impact is likely potentially severe.

We also assumed that the cinereous vulture maintains the same current standing population (relevant demographic data were unavailable) as well as having the same space use in future years. We accounted for the former by primarily expressing mortality proportionately, rather than absolutely (numerically). The latter assumption, besides that recently was found valid for other vultures and raptor species [11,57,58], there is strong evidence from annual comparisons in our study area that is valid also for cinereous vulture [42].

Sex and age class are known not to affect the space use of cinereous vulture in our study area [42], but it is recommended that the effect of these two parameters should be evaluated for other case studies or other species.

### 4.3 Poor environmental legislation implementation

Our findings clearly indicated poor implementation of the SEA Directive [24] relevant to Strategic Environmental Assessment, which calls for the integration of ecological criteria in WPA designation, with emphasis on species of European conservation concern, such as cinereous vulture [32]. The WPA apparently ignored many SPA designations, in contravention of relevant legislation [24,25]. Almost all the WPA fell within the cinereous vulture population range and half of it fell within the species core area, which should be an exclusion zone, since most of the predicted population collision mortality (92%) is expected there.

Spatial planning of the WPA has apparently been performed in the absence of any biological criteria, which is further supported for other species of conservation concern, since the WPA is located in an area that includes all of the few remaining and small colonies of griffon vultures (two in Bulgaria and three in Greece) of Eastern Rhodope, more than half of the few remaining breeding pairs of globally endangered Egyptian vultures (*Neophron percnopterus)* in Greece [54], while it constitutes a major migration corridor and pre-migration concentration area for the Egyptian vultures [59,60] and 60% of golden eagles *Aquila chrysaetos* nests (Sidiropoulos L. *pers*. *comm*.). Besides the high number of raptors colliding with wind turbines (17 carcasses), wind turbines have a clear negative impact of bats as well [61]. We emphasize therefore the necessity for a thorough and rapid update of SEA at national level, incorporating biological criteria and employing a multi-species approach, which would probably completely or largely exclude the current WPA [36] as it stands [33].

Our results, moreover, underlined the poor implementation of environmental impact assessment studies and more stringent assessment obligations under Natura 2000 Directives, since no potentially harmful projects have been identified in practice by any such “assessments”. No wind farm proposals in our study area has been rejected so far on environmental terms, and so several wind farms have been erroneously established in the core area of cinereous vulture and SPAs designated for its protection, causing significant losses to the population [20]. This gives no assurance whatsoever that the many further wind farm proposals will be properly assessed, despite clear legal requirements to do so. This adds further alarm when the greater part (45%) of many hundreds of proposed turbines may be established in the vultures’ core area, with only a small part (8%) in the periphery, where population losses are minimized (Table 1 and Fig 2). The low quality of such assessment studies, effectively authorizing projects with serious negative environmental impacts even inside Natura 2000 sites, applies not only to our study area [62] but elsewhere in Europe [29–31], leading to an urgent call for better control of their quality and implementation [63].

### 4.4 Vulture population at risk

The worst-case scenario assumes that all wind farms in the planning system are authorized and are operating simultaneously, while cinereous vultures maintain the same space use patterns over time. Under this scenario, the vulture population is at immediate risk of persistence, due to the high number of wind turbines and their proposed spatial configuration. When adopting the suggested avoidance rate of 98% for cinereous vulture [20], the population would be rapidly depleted (79% of the standing population gone per year), whilst even under the most optimistic adoption of 99.5% avoidance rate the population decline would be strong but slower (27% of the standing population lost per year) (S4 Table). Regardless of the avoidance rates used, in all cases the models allowed the concrete conclusion that the proposed wind farm developments will likely compromise any possibility of population viability, or existence, within the timeframe of those prospective developments’ planning lifespan (i.e. 25 years).

A more realistic scenario, however, may expect fewer turbines to receive authorization to operate (16% rejected, according to records 2003–2013: 1486 turbines after this assumed rejection rate). Furthermore, we could consider approximately 10% reduction of predicted annual cumulative mortality for this scenario, given the overestimation produced at the coarse conservation zone level (extrapolating for the total number of turbines). This more realistic scenario would still account for a large part of annual population loss (39% of the standing population). Even under the most optimistic but improbable scenario that no further proposals are authorized once the national target is met for the study area, the cumulative collision mortality would still be high (17% of the standing population). Although a population viability analysis, integrating the predicted mortalities within baseline species demographics, would allow a more accurate population trajectory [13,15], our results clearly underline that even under the most optimistic scenario the cinereous vulture is under very serious threat of extinction in the Balkans due to burgeoning wind farm developments.

### 4.5 Conservation-oriented spatial planning

Our findings emphasized the need of officially designating the population core area as a wind farm exclusion zone, since it is the most vital area for the population survival [6], and it was found to account for almost all cumulative collision mortality for cinereous vulture (Table 2 and S4 Table), while also being important for other species prone to collision [20].

We further provided a solution to the spatial planning problem, suggesting the authorization of the proposed wind farms first in the periphery and second in the outer zone of the non-core area, while excluding the other zones. This win-win solution allows harnessing 1095 MW of wind power, readily meeting and even exceeding the national target for the study area, while minimizing the collision mortality effect to the vulture population to only one death per year from prospective future developments. To achieve this minimal 1% population loss per year, collision mortality should be eliminated in the population core area, either by the displacement [13,64] of all currently operating wind turbines in the core area to the periphery, or alternatively by implementing a suite of conservation measures minimizing or eliminating collision mortality [18,20]. For instance, a ‘shutdown on demand’ program of the dangerous turbines would be beneficial for many raptors susceptible to collision. Such a program had some success for griffon vultures in Spain reducing griffon vulture mortality by 50% with only a 0.07% drop in total energy production of the wind farms per year [18]. Our spatial solution would contribute to the long-term survival of cinereous vulture population in the Balkans and safeguard former breeding grounds in Bulgaria [4, 7], providing that international scientific and administrative coordination for Natura 2000 network would be developed in order to implement conservation policies at the appropriate scale [65,66].

## 5. Conclusions

Our research has revealed the magnitude of a potential conflict between large-scale industrial wind-farm developments as an environmentally friendly strategy to combat climate change on the one hand, but on the other hand its detrimental impact on a protected species of conservation concern, when relevant protective legislation is poorly implemented. We have shown that if all wind farms (or the majority, based on recent planning decisions) are to be authorized in south-eastern Europe, the cinereous vulture population is at great risk of extinction in the near future. On prior evidence, proper environmental assessments of wind farms are not expected to hamper authorization of many future harmful developments. Our results provide strong evidence for the official designation of the cinereous vulture population core area as a wind farm exclusion zone and suggest new spatial planning for future wind farm establishment, whereby the national target for wind energy harnessing would be met, while minimising vulture deaths through collision. Our study is a typical example of problem-solving in conservation biology, when full scientific knowledge is lacking, a crisis discipline that calls for immediate conservation decisions, while minimizing the error that stems from incomplete data and/or inherent uncertainties related to the target species’ ecology and behaviour [67,68]. Our methodological approach could be widely adopted as a fast and relatively sound tool for sustainable spatial planning of development projects in a wide suite of similar conflicts relevant to cumulative effects on wildlife populations where site-specific details are absent but wider knowledge of species ranging behaviour is, at least, known crudely.

## Supporting information

S1 Table Technical characteristics and coordinates of the operating and proposed wind farms in the study area. (DOCX)

S2 Table Vulture telemetry locations used in the analysis. (DOCX)

S3 Table Average values per conservation zone of the technical and biological parameters feeding the Collision Risk Model. (DOCX)

S4 Table Predicted cumulative annual collision mortality under different avoidance rates for operating and proposed wind farms per conservation zones. (DOCX)
