# Supplementary material for: A balanced solution to the cumulative threat of industrialized wind farm development on cinereous vultures (Aegypius monachus) in south-eastern Europe
Source: PLoS One. 2017 Feb 23;12(2):e0172685. doi: 10.1371/journal.pone.0172685 (PMC5322877; doi:10.1371/journal.pone.0172685)
Supplement: S1 Table — (DOCX) [file pone.0172685.s001.docx]

**S1 Table.** Technical characteristics and coordinates (geometric centre) of the 155 wind farms (Greece and Bulgaria) included in the analysis of collision mortality estimation for cinereous vulture.

N: Number of wind farm, AS: Authorization stage 1: submission, 2: technical approval, 3: environmental approval. 4: operating. Tu: Number of turbines, Nb: Number of Blades, ATPo: Average turbine power (MW), P: Total wind farm power (ΜW), R: Average rotor diameter (m), H: Average hub height (m), X:Longitude, Y: Latitude, C: Missing exact turbine coordinates, T: Missing technical characteristics.

|  | Technical characteristics | | | | | | | Coordinates | | Missing Data | |
| --- | --- | --- | --- | --- | --- | --- | --- | --- | --- | --- | --- |
| N | As | Tu | Nb | ATPo | P | R | H | X | Y | C | T |
| **Greece** | | | | | | | | | | | |
| 1 | 1 | 5 | 3 | 2 | 10 | 90 | 78 | 25.8337 | 41.1538 |  |  |
| 2 | 4 | 24 | 3 | 1.3 | 31.2 | 62 | 50 | 25.8424 | 41.1473 |  |  |
| 3 | 2 | 5 | 3 | 0.85 | 4.25 | 58 | 65 | 25.8554 | 41.1247 |  |  |
| 4 | 2 | 3 | 3 | 2 | 6 | 90 | 78 | 25.9409 | 41.2059 |  |  |
| 5 | 4 | 22 | 3 | 0.9 | 19.8 | 52 | 49 | 25.8554 | 41.1698 |  |  |
| 6 | 4 | 13 | 3 | 0.9 | 11.7 | 52 | 49 | 25.9516 | 41.0735 |  |  |
| 7 | 4 | 16 | 3 | 0.9 | 14.4 | 52 | 49 | 25.8704 | 41.1413 |  |  |
| 8 | 4 | 13 | 3 | 2 | 26 | 80 | 80 | 25.9176 | 41.1581 |  |  |
| 9 | 1 | 24 | 3 | 2 | 6 | 90 | 80 | 26.1081 | 41.2092 | • | • |
| 10 | 1 | 12 | 3 |  |  |  |  | 26.0702 | 41.0068 | • | • |
| 11 | 1 | 12 | 3 |  |  |  |  | 26.0672 | 40.9608 | • | • |
| 12 | 1 | 9 | 3 |  |  |  |  | 26.0622 | 40.9762 | • | • |
| 13 | 1 | 13 | 3 |  |  |  |  | 25.9826 | 41.1012 | • | • |
| 14 | 1 | 5 | 3 |  |  |  |  | 25.9353 | 41.1332 | • | • |
| 15 | 1 | 15 | 3 |  |  |  |  | 25.9261 | 41.1315 | • | • |
| 16 | 1 | 4 | 3 |  |  |  |  | 25.9123 | 41.1313 |  | • |
| 17 | 1 | 8 | 3 |  |  |  |  | 26.1482 | 40.9611 | • | • |
| 18 | 1 | 5 | 3 |  |  |  |  | 26.1099 | 40.9698 | • | • |
| 19 | 4 | 5 | 3 | 2.5 | 12.5 | 90 | 80 | 26.0945 | 40.9641 |  |  |
| 20 | 4 | 19 | 3 | 2 | 38 | 80 | 60 | 25.9278 | 41.0915 |  |  |
| 21 | 4 | 12 | 3 | 0.9 | 10.8 | 52 | 49 | 25.8248 | 41.1755 |  |  |
| 22 | 1 | 2 | 3 | 2.3 | 4.6 | 71 | 85 | 25.9231 | 41.1274 | • |  |
| 23 | 4 | 17 | 3 | 2.3 | 39.1 | 75 | 85 | 25.9885 | 41.0551 |  |  |
| 24 | 2 | 11 | 3 | 2 | 22 | 100 | 80 | 25.8873 | 41.0930 |  |  |
| 25 | 2 | 6 | 3 | 1.5 | 9 | 77 | 80 | 25.8993 | 41.1327 | • |  |
| 26 | 1 | 15 | 3 | 2 | 30 | 82 | 85 | 25.9753 | 41.2010 |  |  |
| 27 | 1 | 11 | 3 | 3 | 33 | 90 | 80 | 25.8163 | 41.1067 |  |  |
| 28 | 1 | 20 | 3 | 2 | 40 | 80 | 65 | 25.8142 | 41.1056 |  |  |
| 29 | 1 | 16 | 3 | 2 | 32 | 80 | 61 | 25.8840 | 41.0936 |  |  |
| 30 | 1 | 20 | 3 | 2 | 40 | 71 | 64 | 26.0660 | 41.1509 |  |  |
| 31 | 1 | 12 | 3 | 2 | 24 | 90 | 80 | 26.0521 | 40.9850 |  |  |
| 32 | 1 | 9 | 3 | 0.85 | 7.65 | 58 | 55 | 25.9447 | 41.2162 |  |  |
| 33 | 1 | 12 | 3 | 0.85 | 10.2 | 58 | 55 | 25.8526 | 41.1240 |  |  |
| 34 | 1 | 15 | 3 | 2 | 30 | 90 | 80 | 25.9168 | 41.1965 |  |  |
| 35 | 1 | 12 | 3 | 2 | 24 | 90 | 80 | 25.9205 | 41.1214 |  |  |
| 36 | 1 | 15 | 3 | 2 | 30 | 90 | 80 | 26.0912 | 40.9914 |  |  |
| 37 | 1 | 8 | 3 | 2 | 16 | 90 | 80 | 26.0852 | 40.9515 |  |  |
| 38 | 1 | 6 | 3 | 3 | 18 | 112 | 94 | 26.1035 | 40.9745 | • |  |
| 39 | 1 | 6 | 3 | 3 | 18 | 112 | 94 | 26.1672 | 40.9748 |  |  |
| 40 | 1 | 3 | 3 | 3 | 9 | 112 | 94 | 26.1324 | 40.9595 |  |  |
| 41 | 1 | 3 | 3 | 3 | 9 | 112 | 94 | 26.1600 | 40.9654 |  |  |
| 42 | 1 | 10 | 3 | 3 | 30 | 112 | 94 | 25.8602 | 41.1102 |  |  |
| 43 | 4 | 8 | 3 | 0.8 | 6.4 | 50 | 46 | 25.7903 | 41.2030 |  |  |
| 44 | 2 | 6 | 3 | 1.5 | 9 | 70 | 65 | 25.7950 | 41.1280 |  |  |
| 45 | 2 | 2 | 3 | 2 | 4 | 82 | 78 | 25.8855 | 41.0415 |  |  |
| 46 | 2 | 12 | 3 | 2.5 | 30 | 90 | 80 | 25.9622 | 40.9654 |  |  |
| 47 | 1 | 13 | 3 |  |  |  |  | 25.9890 | 41.3078 | • | • |
| 48 | 1 | 10 | 3 |  |  |  |  | 25.9085 | 41.2642 | • | • |
| 49 | 1 | 15 | 3 |  |  |  |  | 25.9271 | 41.2618 | • | • |
| 50 | 1 | 6 | 3 |  |  |  |  | 25.9156 | 41.2897 | • | • |
| 51 | 1 | 13 | 3 |  |  |  |  | 26.0240 | 40.9353 | • | • |
| 52 | 1 | 12 | 3 |  |  |  |  | 26.0104 | 40.9655 | • | • |
| 53 | 3 | 13 | 3 | 2 | 26 | 90 | 80 | 25.8179 | 41.1918 |  |  |
| 54 | 2 | 3 | 3 | 2.5 | 7.5 | 90 | 80 | 25.9474 | 40.9583 |  |  |
| 55 | 2 | 5 | 3 | 2 | 10 | 82 | 59 | 25.8238 | 41.1382 |  |  |
| 56 | 1 | 23 | 3 | 1 | 23 | 58 | 69 | 25.8480 | 41.0908 |  |  |
| 57 | 1 | 6 | 3 | 1.5 | 9 | 77 | 80 | 25.8607 | 41.2130 |  |  |
| 58 | 2 | 9 | 3 | 2 | 18 | 71 | 64 | 25.7869 | 41.1721 |  |  |
| 59 | 1 | 17 | 3 | 2 | 34 | 71 | 64 | 25.7617 | 41.1554 |  |  |
| 60 | 2 | 8 | 3 | 2 | 16 | 71 | 64 | 25.9324 | 41.0501 |  |  |
| 61 | 1 | 15 | 3 | 2 | 30 | 90 | 78 | 25.8499 | 41.2079 |  |  |
| 62 | 1 | 12 | 3 | 3 | 36 | 90 | 80 | 25.8573 | 41.0483 |  |  |
| 63 | 1 | 20 | 3 | 2 | 40 | 80 | 79 | 25.8518 | 41.0478 |  |  |
| 64 | 1 | 8 | 3 | 2 | 16 | 90 | 80 | 25.7437 | 41.2117 |  |  |
| 65 | 1 | 16 | 3 | 2 | 32 | 90 | 80 | 26.0908 | 40.8953 |  |  |
| 66 | 1 | 4 | 3 | 3 | 12 | 112 | 94 | 26.1109 | 40.9393 |  |  |
| 67 | 1 | 9 | 3 | 2.3 | 20.7 | 82 | 80 | 25.9122 | 41.0463 |  |  |
| 68 | 1 | 6 | 3 | 2.3 | 13.8 | 82 | 80 | 25.9879 | 41.0327 |  |  |
| 69 | 1 | 17 | 3 | 2 | 34 | 97 | 78 | 25.8723 | 41.2707 |  |  |
| 70 | 4 | 7 | 3 | 1.3 | 9.1 | 62 | 50 | 25.8906 | 41.2887 |  |  |
| 71 | 1 | 3 | 3 | 2 | 6 | 90 | 78 | 25.9027 | 41.2842 |  |  |
| 72 | 1 | 12 | 3 |  |  |  |  | 26.0795 | 40.8710 | • | • |
| 73 | 1 | 3 | 3 |  |  |  |  | 26.0761 | 40.8869 | • | • |
| 74 | 1 | 9 | 3 | 2.3 | 20.7 | 71 | 98 | 25.6832 | 41.1865 |  |  |
| 75 | 1 | 10 | 3 | 2 | 20 | 90 | 80 | 25.6831 | 41.1844 |  |  |
| 76 | 1 | 8 | 3 | 2 | 16 | 90 | 80 | 25.7016 | 41.1624 |  |  |
| 77 | 1 | 13 | 3 | 2 | 26 | 90 | 80 | 25.7184 | 41.1965 |  |  |
| 78 | 1 | 11 | 3 | 2 | 22 | 90 | 80 | 25.6810 | 41.1732 |  |  |
| 79 | 1 | 14 | 3 | 3 | 42 | 90 | 80 | 26.0070 | 40.9092 |  |  |
| 80 | 1 | 1 | 3 | 2.6 | 2.6 | 100 | 94 | 25.9637 | 40.9262 |  |  |
| 81 | 1 | 70 | 3 | 3 | 210 | 82 | 78 | 25.6621 | 41.1915 |  |  |
| 82 | 1 | 12 | 3 | 2 | 24 | 97 | 78 | 25.6452 | 41.1524 |  |  |
| 83 | 1 | 13 | 3 | 2 | 26 | 90 | 80 | 25.7486 | 41.2388 |  |  |
| 84 | 1 | 14 | 3 | 2 | 28 | 90 | 80 | 25.8133 | 41.2647 |  |  |
| 85 | 4 | 24 | 3 | 1.3 | 31.2 | 62 | 50 | 25.8712 | 41.3051 |  |  |
| 86 | 1 | 10 | 3 |  |  |  |  | 25.9048 | 41.3035 | • | • |
| 87 | 1 | 10 | 3 |  |  |  |  | 25.9301 | 41.3126 | • | • |
| 88 | 1 | 27 | 3 | 2 | 20 | 90 | 80 | 25.7590 | 41.2690 | • |  |
| 89 | 1 | 12 | 3 | 2 | 16 | 90 | 80 | 25.7218 | 41.2624 | • |  |
| 90 | 1 | 22 | 3 | 2 | 44 | 90 | 78 | 25.8370 | 41.3094 |  |  |
| 91 | 2 | 7 | 3 | 2 | 14 | 90 | 78 | 25.8245 | 41.2976 |  |  |
| 92 | 1 | 14 | 3 | 3 | 42 | 90 | 80 | 25.6654 | 41.2191 |  |  |
| 93 | 1 | 14 | 3 | 2 | 28 | 90 | 80 | 25.7295 | 41.2436 |  |  |
| 94 | 1 | 11 | 3 | 3 | 33 | 90 | 80 | 25.6385 | 41.1853 |  |  |
| 95 | 1 | 8 | 3 | 2 | 16 | 90 | 80 | 25.6826 | 41.1972 |  |  |
| 96 | 1 | 18 | 3 | 2 | 36 | 90 | 80 | 25.8065 | 41.2829 |  |  |
| 97 | 1 | 23 | 3 | 2 | 46 | 90 | 78 | 25.7072 | 41.2952 |  |  |
| 98 | 1 | 5 | 3 | 2 | 10 | 90 | 78 | 25.6949 | 41.2412 |  |  |
| 99 | 1 | 26 | 3 | 2 | 24 | 82 | 78 | 25.7497 | 40.9073 | • |  |
| 100 | 1 | 9 | 3 |  |  |  |  | 25.7462 | 40.9365 | • | • |
| 101 | 1 | 12 | 3 |  |  |  |  | 25.7463 | 40.9218 | • | • |
| 102 | 1 | 10 | 3 |  |  |  |  | 25.6189 | 41.2616 | • | • |
| 103 | 1 | 16 | 3 |  |  |  |  | 25.5918 | 41.2711 | • | • |
| 104 | 1 | 7 | 3 |  |  |  |  | 25.5460 | 41.2285 | • | • |
| 105 | 1 | 4 | 3 |  |  |  |  | 25.5331 | 41.2186 | • | • |
| 106 | 1 | 6 | 3 |  |  |  |  | 25.4938 | 41.1881 | • | • |
| 107 | 1 | 3 | 3 |  |  |  |  | 25.5112 | 41.1753 | • | • |
| 108 | 1 | 1 | 3 | 2 | 2 | 82 | 78 | 25.7518 | 40.8942 |  |  |
| 109 | 4 | 5 | 3 | 0.6 | 3 | 44 | 46 | 25.7430 | 40.8961 |  |  |
| 110 | 2 | 11 | 3 | 2 | 22 | 71 | 64 | 25.6969 | 41.2941 |  |  |
| 111 | 2 | 15 | 3 | 2 | 30 | 90 | 78 | 25.8409 | 41.3284 |  |  |
| 112 | 1 | 23 | 3 | 2 | 46 | 90 | 80 | 26.3399 | 41.3237 |  |  |
| 113 | 1 | 6 | 3 | 3 | 18 | 90 | 80 | 25.4585 | 41.1769 |  |  |
| 114 | 1 | 20 | 3 | 2 | 40 | 90 | 80 | 26.2914 | 41.3019 |  |  |
| 115 | 1 | 16 | 3 | 2 | 32 | 90 | 105 | 26.2968 | 40.9625 |  |  |
| 116 | 1 | 11 | 3 | 2.3 | 25.3 | 71 | 98 | 25.6238 | 41.2038 |  |  |
| 117 | 1 | 13 | 3 | 3 | 39 | 90 | 80 | 25.6172 | 41.2006 |  |  |
| 118 | 1 | 14 | 3 | 3 | 42 | 90 | 80 | 25.6359 | 41.2096 |  |  |
| 119 | 1 | 18 | 3 | 3 | 54 | 90 | 80 | 25.6495 | 41.2787 |  |  |
| 120 | 1 | 1 | 3 | 3 | 3 | 101 | 99 | 26.0902 | 40.8450 |  |  |
| 121 | 1 | 78 | 3 | 4.5 | 351 | 136 | 120 | 25.5176 | 41.1082 |  |  |
| 122 | 1 | 7 | 3 | 2 | 14 | 90 | 78 | 25.5913 | 41.2426 |  |  |
| 123 | 1 | 15 | 3 | 2 | 30 | 90 | 78 | 25.8192 | 41.3369 |  |  |
| 124 | 1 | 10 | 3 | 2 | 20 | 90 | 80 | 25.5280 | 41.2486 |  |  |
| 125 | 1 | 7 | 3 |  |  |  |  | 25.5811 | 41.2808 | • | • |
| 126 | 1 | 4 | 3 |  |  |  |  | 25.5730 | 41.2669 | • | • |
| 127 | 1 | 2 | 3 |  |  |  |  | 25.4757 | 41.2718 | • | • |
| 128 | 1 | 2 | 3 |  |  |  |  | 25.5456 | 40.9650 | • | • |
| 129 | 1 | 4 | 3 |  |  |  |  | 25.5795 | 40.9272 | • | • |
| 130 | 1 | 3 | 3 |  |  |  |  | 25.5933 | 40.9260 | • | • |
| 131 | 1 | 5 | 3 |  |  |  |  | 25.6073 | 40.9115 | • | • |
| 132 | 1 | 3 | 3 | 2 | 6 | 82 | 78 | 25.1134 | 41.2021 |  |  |
| 133 | 1 | 3 | 3 | 2 | 6 | 90 | 80 | 26.3696 | 41.3092 |  |  |
| 134 | 1 | 1 | 3 | 2 | 2 | 90 | 80 | 26.3705 | 41.2980 |  |  |
| 135 | 1 | 6 | 3 | 3 | 18 | 90 | 80 | 25.4606 | 41.2216 |  |  |
| 136 | 1 | 11 | 3 | 2 | 22 | 90 | 80 | 25.4506 | 41.2563 |  |  |
| 137 | 1 | 6 | 3 | 2 | 12 | 90 | 80 | 25.1189 | 41.1959 |  |  |
|  | **Subtotal** | 1580 |  |  | 2744 |  |  |  |  | 40 | 35 |
|  | **Average** | 11.5 | 3 | 2.09 | 26.6 | 85 | 77 |  |  |  |  |
|  | **SD** | 9.75 |  | 0.63 | 39.2 | 15 | 13 |  |  |  |  |
| **Bulgaria** | | | | | | | | | | | |
| 138 | 1 | 7 | 3 |  |  |  |  | 25.9553 | 41.3190 |  | • |
| 139 | 1 | 20 | 3 |  |  |  |  | 25.7090 | 41.4928 |  | • |
| 140 | 1 | 14 | 3 |  |  |  |  | 25.7359 | 41.4961 |  | • |
| 141 | 1 | 10 | 3 |  |  |  |  | 25.7300 | 41.4818 |  | • |
| 142 | 1 | 7 | 3 |  |  |  |  | 25.6927 | 41.4750 |  | • |
| 143 | 1 | 5 | 3 |  |  |  |  | 25.7075 | 41.4763 |  | • |
| 144 | 1 | 14 | 3 |  |  |  |  | 25.6780 | 41.4832 |  | • |
| 145 | 1 | 15 | 3 |  |  |  |  | 25.8817 | 41.5364 |  | • |
| 146 | 1 | 2 | 3 |  |  |  |  | 25.8560 | 41.5479 |  | • |
| 147 | 1 | 1 | 3 |  |  |  |  | 25.8739 | 41.5399 |  | • |
| 148 | 1 | 1 | 3 |  |  |  |  | 25.6749 | 41.4716 |  | • |
| 149 | 1 | 5 | 3 |  |  |  |  | 25.8687 | 41.3271 |  | • |
| 150 | 1 | 7 | 3 |  |  |  |  | 25.8337 | 41.3425 |  | • |
| 151 | 1 | 4 | 3 |  |  |  |  | 25.8525 | 41.3459 |  | • |
| 152 | 1 | 4 | 3 |  |  |  |  | 25.8191 | 41.3599 |  | • |
| 153 | 1 | 6 | 3 |  |  |  |  | 25.8216 | 41.3950 |  | • |
| 154 | 1 | 5 | 3 |  |  |  |  | 25.7660 | 41.3283 |  | • |
| 155 | 1 | 5 | 3 |  |  |  |  | 25.8616 | 41.5637 |  | • |
|  | **Subtotal** | 132 |  |  |  |  |  |  |  |  | 18 |
|  | **Average** | 7.33 | 3 |  |  |  |  |  |  |  |  |
|  | **SD** | 5.26 |  |  |  |  |  |  |  |  |  |
| **Total** |  | **1712** | **3** |  | **2744** |  |  |  |  | **40** | **53** |
| **Average** |  |  |  | **2.09** | **26.6** | **85** | **77** |  |  |  |  |
| **SD** |  |  |  | **0.63** | **39.2** | **15** | **13** |  |  |  |  |
