# Supplementary material for: A balanced solution to the cumulative threat of industrialized wind farm development on cinereous vultures (Aegypius monachus) in south-eastern Europe
Source: PLoS One. 2017 Feb 23;12(2):e0172685. doi: 10.1371/journal.pone.0172685 (PMC5322877; doi:10.1371/journal.pone.0172685)
Supplement: S2 Table — (DOCX) [file pone.0172685.s002.docx]

**S2 Table** Dataset of locations used in the analysis from the seven GPS-tagged cinereous vultures tracked for the period 2007 to 2009 (Vassilakis et al. 2016). GPS-Plus transmitters (Vectronic Aerospace GmbH, Berlin, Germany) recorded vultures’ locations randomly three times per day during daytime, and every hour for two random days per month. Telus-Mini transmitters (Televilt/TVP-Positioning, Lindesberg, Sweden) recorded locations every 45 minutes, during daytime.

| **N** | **Individual vulture code** | **Tag type** | **Number of locations** | **Monitoring period** | | | **Tracking duration (months)** | | | |  |
| --- | --- | --- | --- | --- | --- | --- | --- | --- | --- | --- | --- |
|  |  |  |  |  |  |  | **Age classes** | | | **Total** | |
|  |  |  |  | **Start** | **End** | **Ns** | **J** | **I** | **A** |  |  |
| 1 | 113H88 | GPS Plus | 494 | 1/2/2007 | 31/8/2008 | 3 | – | 12.0 | 7.0 | 19.0 | |
| 2 | 3664H71 | GPS Plus | 1023 | 1/2/2008 | 31/3/2009 | 3 | – | 12.0 | 2.0 | 14.0 | |
| 3 | 3688H38 | GPS Plus | 982 | 1/2/2008 | 31/3/2009 | 3 | – | – | 14.0 | 14.0 | |
| 4 | 2021H83 | Telus-Mini | 3506 | 14/10/2008 | 21/4/2009 | 2 | – | – | 6.1 | 6.1 | |
| 5 | 2022H67 | Telus-Mini | 3027 | 14/10/2008 | 10/4/2009 | 2 | 3.5 | 2.3 | – | 5.8 | |
| 6 | 2023H45 | Telus-Mini | 2448 | 31/10/2008 | 21/3/2009 | 2 | – | 4.6 | – | 4.6 | |
| 7 | 2024H56 | Telus-Mini | 3342 | 31/10/2008 | 10/5/2009 | 2 | – | 6.3 | – | 6.3 | |
| **Average** | – | – | 2117 | – | – | 2.4 | – | 7.4 | 7.3 | 10.0 | |
| **SD** | – | – | 1257 | – | – | 0.5 | – | 4.4 | 5.0 | 5.6 | |
| **Total** | – | – | 14822 | – | – | 17.0 | 3.5 | 37.2 | 29.1 | 69.8 | |

N: Number of Individuals, Ns: Number of season (breeding/non breeding), F: Fledgling, J: Juvenile (2^nd^ Calendar Year; CY), I: Immature (3^rd^-4^th^ CY), A: Adult (≥5^th^ CY).
