# Supplementary material for: A balanced solution to the cumulative threat of industrialized wind farm development on cinereous vultures (Aegypius monachus) in south-eastern Europe
Source: PLoS One. 2017 Feb 23;12(2):e0172685. doi: 10.1371/journal.pone.0172685 (PMC5322877; doi:10.1371/journal.pone.0172685)
Supplement: S3 Table — (DOCX) [file pone.0172685.s003.docx]

**S3 Table** Average values per conservation zone of the technical and biological parameters feeding the Collision Risk Model (CRM).

| **Conservation zones** | |  | **Technical parameters** | | | | | | | | | | |  | **Biological parameters** | | |
| --- | --- | --- | --- | --- | --- | --- | --- | --- | --- | --- | --- | --- | --- | --- | --- | --- | --- |
|  | **Code** |  | **P**  **(MW)** | **Tu** | **Mt (%)** | **Ml**  **(%)** | **ATPo (MW)** | **H (m)** | **R (m)** | **Ch (m)** | **Rp (s)** | **Rz (m)** | **A (km²)** |  | **N** | **T1 (%)** | **T2 (%)** |
| **Core area** | 4 |  | 500 | 365 | 21.6 | 30.4 | 1.9 | 71 | 80 | 3.50 | 2.95 | 31.46m to 111.49m | 34.84 |  | 17 | 33.30 | 65.09 |
|  | 3 |  | 478 | 304 | 14.5 | 14.5 | 1.8 | 70 | 76 | 3.33 | 2.80 | 32.25m to 108.15m | 27.06 |  | 12 | 32.25 | 21.30 |
|  | 2 |  | 244 | 135 | 14.1 | 13.3 | 2.1 | 75 | 83 | 3.63 | 3.06 | 33.44m to 116.49m | 13.31 |  | 7 | 32.34 | 7.19 |
|  | 1 |  | – | 63 | 100.0 | – | – | 72* | 80* | 3.49* | 2.93* | 32.50m to 111.50m | 4.75 |  | 2 | 32.63 | 0.31 |
|  | Average |  | 408 | 217 | 37.5 | 19.4 | 1.9 | 72 | 80 | 3.49 | 2.93 | – | 19.99 |  | 10 | 32.63 | 23.47 |
|  | SD |  | 142 | 141 | 41.8 | 9.5 | 0.1 | 2 | 4 | 0.15 | 0.13 | – | 13.51 |  | 6 | 0.48 | 29.09 |
|  | Total |  | 1 223 | 867 | 23.6 | 20.0 | – | – | – | – | – | – | 79.95 |  | – | – | – |
| **Non-core area** | 4 |  | 4 | 4 | 25.0 | 25.0 | 1.2 | 63 | 69 | 3.34 | 3.56 | 29m to 97.67m | 0.65 |  | 17 | 33.81 | 0.86 |
|  | 3 |  | 46 | 40 | 42.5 | 42.5 | 2.0 | 80 | 91 | 3.64 | 3.35 | 34.28m to 125.20m | 4.53 |  | 12 | 32.76 | 3.20 |
|  | 2 |  | 377 | 225 | 15.1 | 32.0 | 2.2 | 78 | 87 | 3.48 | 3.20 | 34.45m to 121.87m | 21.55 |  | 7 | 32.18 | 5.42 |
|  | 1 |  | 888 | 460 | 25.9 | 19.8 | 2.7 | 86 | 94 | 3.76 | 3.46 | 39.15m to 133.28m | 42.16 |  | 2 | 29.43 | 1.35 |
|  | Average |  | 329 | 182 | 27.1 | 29.8 | 2.0 | 77 | 85 | 3.55 | 3.39 | – | 17.22 |  | 10 | 32.05 | 2.70 |
|  | SD |  | 408 | 209 | 11.4 | 9.8 | 0.6 | 10 | 11 | 0.18 | 0.15 | – | 18.94 |  | 6 | 1.87 | 2.07 |
|  | Total |  | 1 314 | 729 | 23.5 | 24.8 | – | – | – | – | – | – | 68.88 |  | – | – | – |
| **Periphery** |  |  | 207 | 116 | 34.5 | 31.0 | 2.7 | 89 | 101 | 4.12 | 3.83 | 38.79m to 139.37m | 13.25 |  | 3 | 30.26 | 0.44 |
| **Average** |  |  | 343 | 190 | 32.6 | 26.1 | 2.0 | 86 | 78 | 3.60 | 3.28 | – | 18.01 |  | 9 | 32.11 | 11.68 |
| **SD** |  |  | 286 | 158 | 27.0 | 9.9 | 0.4 | 19 | 16 | 0.26 | 0.34 | – | 14.42 |  | 6 | 1.40 | 21.08 |
| **Grand Total** | |  | 2 744 | 1 712 | 24.3 | 22.8 | – | – | – | – | – | – | 162.09 |  | – | – | – |

Code: 1: 1–4 individuals, 2: 5–9 individuals, 3: 10–14 individuals, 4: 15–19 individuals, Periphery: 1–6 individuals, P: Total wind farm power, Tu: Number of turbines, Mt: Percentage of turbines with missing technical characteristics, Mc: Percentage of turbines with missing coordinates, ATPo: Average turbine power, H: Average hub height, R: Average rotor diameter, Ch: Average ax chord, Rp: Average rotation period, Rz: Rotor risk zone, A: Wind farm area (200 m buffer around turbines ), N: Number of vultures that used each conservation zone (median value), T1: Time flying at Rz, T2: Time spent by the population in wind farm area. * Average values of core area used due to missing data.
