# Supplementary material for: A balanced solution to the cumulative threat of industrialized wind farm development on cinereous vultures (Aegypius monachus) in south-eastern Europe
Source: PLoS One. 2017 Feb 23;12(2):e0172685. doi: 10.1371/journal.pone.0172685 (PMC5322877; doi:10.1371/journal.pone.0172685)
Supplement: S4 Table — (DOCX) [file pone.0172685.s004.docx]

**S4 Table** Predicted additive and cumulative annual collision mortality under the 95%, 98% and 99.5% avoidance rates for operating and proposed wind farms per conservation zones. See Fig 2 for conservation zone and Table 2 for mortality under the 99% avoidance rate.

|  |  | **Avoidance rate 95%** | | | | | | | | | **Avoidance rate 98%** | | | | | | | | | **Avoidance rate 99,5%** | | | | | | | | |
| --- | --- | --- | --- | --- | --- | --- | --- | --- | --- | --- | --- | --- | --- | --- | --- | --- | --- | --- | --- | --- | --- | --- | --- | --- | --- | --- | --- | --- |
|  |  | **Operating** | | | | | **Proposed** | | | | **Operating** | | | | | **Proposed** | | | | **Operating** | | | | | **Proposed** | | | |
|  | **Zones** | **C** | **C (%)** | **C_A_** | **CC_Z_** | **CC_W_** | **C** | **C (%)** | **C_A_** | **CC_Z_** | **C** | **C (%)** | **C_A_** | **CC_Z_** | **CC_W_** | **C** | **C (%)** | **C_A_** | **CC_Z_** | **C** | **C (%)** | **C_A_** | **CC_Z_** | **CC_W_** | **C** | **C (%)** | **C_A_** | **CC_Z_** |
| **Core area** | 4 | 18.80 | 67.27 | 18.80 | 18.80 | 17.36 | 191.06 | 62.46 | 191.06 | 191.06 | 7.52 | 67.27 | 7.52 | 7.52 | 7.29 | 76.42 | 62.46 | 76.42 | 76.42 | 1.88 | 67.27 | 1.88 | 1.88 | 1.87 | 19.11 | 62.46 | 19.11 | 19.11 |
|  | 3 | 8.08 | 28.89 | 26.88 | 25.41 | 23.7 | 63.90 | 20.89 | ** | ** | 3.23 | 28.89 | 10.75 | 10.52 | 10.24 | 25.56 | 20.89 | 101.98 | 83.02 | 0.81 | 28.89 | 2.69 | 2.67 | 2.66 | 6.39 | 20.89 | 25.50 | 24.31 |
|  | 2 | 0.63 | 2.27 | 27.51 | 25.87 | 24.17 | 20.07 | 6.56 | – | – | 0.25 | 2.27 | 11.01 | 10.74 | 10.47 | 8.03 | 6.56 | 110.01 | 83.10 | 0.06 | 2.27 | 2.75 | 2.73 | 2.72 | 2.01 | 6.56 | 27.50 | 25.82 |
|  | 1 | 0.00 | – | 27.51 | 25.87 | 24.17 | 1.13 | 0.37 | – | – | 0.00 | – | 11.01 | 10.74 | 10.47 | 0.45 | 0.37 | 110.46 | 83.07 | 0.00 | – | 2.75 | 2.73 | 2.72 | 0.11 | 0.37 | 27.62 | 25.90 |
|  | Total | 27.51 | 98.42 | 27.51 | 25.87 | 24.17 | 276.16 | 90.29 | – | – | 11.01 | 98.42 | 11.01 | 10.74 | 10.47 | 109.68 | 90.29 | 110.46 | 83.07 | 2.75 | 98.42 | 2.75 | 2.73 | 2.72 | 27.62 | 90.29 | 27.62 | 25.90 |
| **Non core area** | 4 | – | – | 27.51 | 25.87 | 24.17 | 1.80 | 0.59 | – | – | – | – | 11.01 | 10.74 | 10.47 | 0.72 | 0.59 | 111.18 | 83.02 | 0.00 | 0.00 | 2.75 | 2.73 | 2.72 | 0.18 | 0.59 | 27.80 | 26.04 |
|  | 3 | – | – | 27.51 | 25.87 | 24.17 | 7.89 | 2.58 | – | – | – | – | 11.01 | 10.74 | 10.47 | 3.16 | 2.58 | 114.34 | 82.76 | 0.00 | 0.00 | 2.75 | 2.73 | 2.72 | 0.79 | 2.58 | 28.58 | 26.61 |
|  | 2 | 0.18 | 0.18 | 27.70 | 26.01 | 24.3 | 15.29 | 5.00 | – | – | 0.07 | 0.65 | 11.08 | 10.81 | 10.53 | 6.12 | 5.00 | 120.45 | 82.09 | 0.02 | 0.65 | 2.77 | 2.75 | 2.74 | 1.53 | 5.00 | 30.11 | 27.72 |
|  | 1 | 0.26 | 0.26 | 27.96 | 26.20 | 24.49 | 3.73 | 1.22 | – | – | 0.10 | 0.93 | 11.18 | 10.90 | 10.63 | 1.49 | 1.22 | 121.95 | 81.84 | 0.03 | 0.93 | 2.80 | 2.78 | 2.76 | 0.37 | 1.22 | 30.49 | 27.98 |
|  | Total | 0.44 | 0.44 | 27.96 | 26.20 | 24.49 | 28.71 | 9.39 | – | – | 0.18 | 1.58 | 11.18 | 10.90 | 10.63 | 11.96 | 9.39 | 121.95 | 81.84 | 0.04 | 1.58 | 2.80 | 2.78 | 2.76 | 2.87 | 9.39 | 30.49 | 27.98 |
| **Periphery** |  | – | – | 27.96 | 26.20 | 24.49 | 1.01 | 0.33 | – | – | – | – | 11.18 | 10.90 | 10.63 | 0.40 | 0.33 | 122.35 | 81.76 | 0.00 | – | 2.80 | 2.78 | 2.76 | 0.10 | 0.33 | 30.59 | 28.05 |
| **Grand Total** | | 27.96 | 98.86 | 27.96 | 26.20 | 24.49 | 305.87 | 100.00 | – | – | 11.18 | 100.00 | 11.18 | 10.90 | 10.63 | 122.32 | 100.00 | 122.35 | 81.76 | 2.80 | 100.00 | 2.80 | 2.78 | 2.76 | 30.59 | 100.00 | 30.59 | 28.05 |

Zones: 1: 1–4 individuals, 2: 5–9 individuals, 3: 10–14 individuals, 4: 15–19 individuals, Periphery: 1–6 individuals, C: Collisions per year (individuals), C(%): Percentage of annual collisions, C_A_: Annual additive collisions, CC_Z_: Annual cumulative collisions considering all the wind farms per zone as one mega wind farm, CC_W_: Cumulative annual collisions considering the operating wind farms on a case-by-case basis. **The population goes extinct.
